# Daytime Sleepiness in Parkinson's Disease: Perception, Influence of Drugs, and Mood Disorder

**DOI:** 10.1155/2014/939713

**Published:** 2014-01-22

**Authors:** M. Ataide, C. M. R. Franco, O. G. Lins

**Affiliations:** ^1^Pós-Graduação em Neuropsiquiatria e Ciências do Comportamento, Universidade Federal de Pernambuco, Recife, PE, Brazil; ^2^Hospital das Clinicas, Universidade Federal de Pernambuco, Recife, PE, Brazil

## Abstract

Parkinson's disease (PD) is associated with sleep complaints as excessive daytime sleepiness (EDS) and several factors have been implicated in the genesis of these complaints. *Objective*. To correlate the subjective perception of EDS with variables as the severity of the motor symptoms, medications, and the presence of depressive symptoms. *Materials and Methods*. A cross-sectional study, using specific scales as Epworth sleepiness scale (ESS), Beck depression inventory (iBeck) and Hoehn and Yahr (HY), in 42 patients with PD. *Results*. The patients had a mean age of 61.2 ± 11.3 years and mean disease duration of 4.96 ± 3.3 years. The mean ESS was 7.5 ± 4.7 and 28.6% of patients reached a score of abnormally high value (>10). There was no association with gender, disease duration, and dopamine agonists. Patients with EDS used larger amounts of levodopa (366.7 ± 228.0 versus 460.4 ± 332.25 mg, *P* = 0.038), but those who had an iBeck >20 reached lower values of ESS than the others (5.9 ± 4.1 versus 9.3 ± 4.8, *P* = 0.03). *Conclusions*. EDS was common in PD patients, being related to levodopa intake. Presence of depressed mood may influence the final results of self-assessment scales for sleep disorders.

## 1. Introduction

Parkinson's disease (PD) is a leading progressive neurodegenerative disease, with prevalence estimated 1-2% of the population above 55 years. Sleep-related complaints are frequent in this population and, in some cases, may be the initial manifestation of the disease. Around 60 to 90% of PD patients affected by sleep disorders suffer negative impact on their quality of life [1]. A population study, which evaluated 245 patients with Parkinson's disease, showed that more than two-thirds of them had complaints about sleep disturbances and complaints of the same type are found in 46% of diabetic patients and 33% of control patients [2].

Excessive daytime sleepiness (EDS) has an estimated prevalence from 15.5 to 74% of PD patients [1]. Clinical evidence support the hypothesis of EDS being a particular symptom of PD and its potential association with disease progression [2–4]. However, there are studies that contradict this association [5–7]. The EDS can also arise as a secondary symptom nighttime sleep deprivation or other sleep disorders such as sleep apnea (present in 20–30% of PD patients). Patients with REM sleep behavior disorders did not show greater EDS, even if REM sleep is interrupted by violent dreams [8]. Finally, the association between the dopamine replacement therapy and the EDS has been described [1]. Although there are reports that the dopamine agonists cause drowsiness as a class effect [9], in many studies, the main predictive factor is the total amount of the dose dopamine [10, 11].

Several subjective measures have been proposed to assess EDS in PD. By using the Epworth sleepiness scale (ESS) [12], patients with scores higher than 7 show a sensitivity of 75% risk of road accidents [4]. Some studies have indicated that the ESS shows a correlation with objective tests, such as the Multiple Sleep Latency Test (MSLT) [5, 10, 13], while others did not show this correlation [14, 15]. Nevertheless, the ESS is recommended, by the Movement Disorders Society, for the evaluation of EDS in patients with PD and it has been proposed that the cut-off of 10/11 is indicative of pathological sleepiness [16].

In relation to mood disorders, depression is very common in PD patients and it is a major cause of insomnia in this population [17], but its relationship with EDS is questionable.

The objective of this study is to evaluate, through a subjective measure of the level of daytime sleepiness, the characteristics and determinants of EDS in patients with PD, including the influence of depressive symptoms.

## 2. Materials and Methods

This is an observational study conducted in the outpatient clinic of Neurology, in the Hospital das Clinicas of Universidade Federal de Pernambuco, Brazil, from January 2011 to August 2012. All patients gave their written informed consent to participate in the study, which was approved by the ethics committee in research involving humans at the Center for Health Sciences of Universidade Federal de Pernambuco.

Forty-two patients fulfilling clinical criteria of PD (the United Kingdom Parkinson's Disease Society Brain Bank clinical diagnostic criteria) were included in the study. Cognition was evaluated by Mini-Mental State Examination (MMSE). Patients had to have an MMSE score equal to or above 24.

Patients in the study answered a questionnaire that includes disease duration and drug record. To compare different medications directly at dosages of equivalent efficacy, we converted the dosages into levodopa dosage equivalents (LDEs) [18]. The following formula was used: LDE = (regular levodopa dose × 1) + (levodopa controlled release dose × 0.75) + (pramipexole dose × 67) + (ropinirole dose × 16.67) + (pergolide dose × 100) + (bromocriptine dose × 10) + {[regular levodopa dose + (levodopa controlled release dose × 0.75)] × 0.25 if taking tolcapone}. PD symptoms were evaluated using Hoehn and Yahr modified version (HY). To assess depressive symptoms we used the Beck depression inventory (iBeck). In the subjective assessment of daytime sleepiness, we used ESS, considering cut-off from 10 points as the presence of pathological sleepiness.

The *Statistica* (data analysis software system) for Windows version 8.0 (2007) was used for all analyses. Descriptive statistics were used as required. Since most parameters did not follow a normal distribution, nonparametric tests were applied, such as the Mann-Whitney test. Spearman's rank correlation coefficients were used to determine the association between ESD and other variables, such as disease duration, motor and depression symptoms, and use of antiparkinsonian medications. Fisher's exact test was used for dichotomous variables. Significance was defined as *P* < 0.05.

## 3. Results

Of a total of 65 patients interviewed, seven were excluded because they did not meet the diagnostic criteria for idiopathic PD and sixteen, because they had cognitive impairment that would hinder the completion of the scales and questionnaire. Demographic and clinical characteristics are shown in Table 1. The duration of parkinsonian symptoms was 4.96 ± 3.3 (mean ± standard deviation) years (range: 1–14). The scores of motor symptoms, according to the H&Y, were 2.1 ± 0.98 points (range: 1–4). Twelve patients were in advanced stages of PD (HY ≥ 3) and five were in stage 4. The mean Mini-Mental State Examination was 26.7 ± 2.6 points (range: 24–30). All patients were taking antiparkinsonian drugs: levodopa (*n* = 37), pramipexole (*n* = 23), amantadine *n* = 8 (*n* = 8), and biperiden (*n* = 7). The mean levodopa equivalent dose (LED) was 441.3 ± 272.5 mg. Ten patients (24%) were taking benzodiazepines and/or antidepressants. Regarding depressive symptoms, the average score was 18.7 ± 10.7 points (range = 0–42) and 22 (52.4%) patients had moderate to severe depressive symptoms.

The mean ESS was 7.5 ± 4.7 points (range = 0–19), with a median of 7 points. Twelve (28.6%) patients had excessive daytime sleepiness (ESS score > 10 points). There were not significant differences in age, gender, disease duration, motor symptoms, and levodopa equivalent dose between patients with and without EDS. The patients with EDS showed use of higher doses of levodopa than the patients without EDS (460.4 ± 332.25 versus 366.7 ± 228.0 mg, *P* = 0.038). However, when evaluating patients using levodopa alone and levodopa with pramipexole, there was not increase in sleepiness with the addition of dopamine agonist (Fisher's exact test, *P* = 0.50). To the remaining variables, we did not observe any association with pathological sleepiness, including the use of benzodiazepines and antidepressants, where increased drowsiness with use of these medications was not observed (Fisher's exact test, *P* = 0.20) (Table 2).

**Figure 1:**
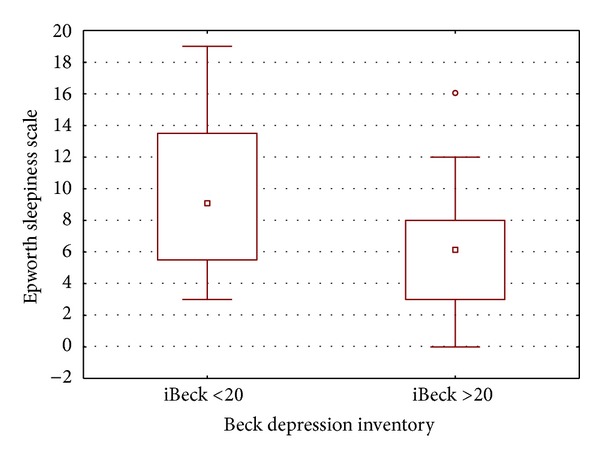
Association of daytime sleepiness, by the Epworth sleepiness scale, and depressive symptoms, as the Beck depression inventory.

There was a trend for lower depressive symptoms scores, through iBeck, in patients with EDS than those without (13.8 ± 9 versus 20.6 ± 10.9, *P* = 0.056), showing a weak association between these two variables (*r*_*s*_ = −0.32). Twenty-two patients had scores iBeck > 20. These patients with more depressive symptoms had lower levels of ESE compared to patients with iBeck scores ≤ 20 (5.9 ± 4.1  versus  9.3 ± 4.8, *P* = 0.03) (Figure 1).

## 4. Discussion

The data demographic of this study is presented in accordance with those found in world literature. Thus, although the males have predominated in the screening evaluation, the females have prevailed in the interviews, a fact possibly related to increased attendance and interest in participating in this gender.

Excessive daytime sleepiness occurred in 28.2% of patients with a mean score of ESS similar to those found in other studies, within an interval ranging from 4.9 ± 3.6 [19] to 11.1 ± 5.9 [20]. Margis et al., in a study in the Brazilian population, found a mean score of 7.74 ± 4.82 [21]. However, some considerations must be made when using the ESS. To be a self-assessment scale, the interpretation of the items is linked with the sociocultural and linguistic characteristics of each population, so that the comparison between studies is valid, but the comparison within the same population may be more realistic. Santamaria already commented on the ambiguity of some items of ESS [22]. Study objectives, such as the MSLT, can provide more concrete and less risk of bias on this aspect.

In this study, EDS was associated with the total amount of levodopa. One of the first studies that evaluated the chronic use of levodopa showed that daytime sleepiness was disabling adverse effects in 13.7% of patients [23] and that the patients became sleepy more after one year of drug treatment [24]. Kaynak et al., who used the MSLT for evaluation of EDS, have shown that daytime sleepiness was not present in untreated patients but appeared later during dopaminergic treatment and that high dose of levodopa was a strong predictor of EDS [25].

However, in our study it was not observed significant association between EDS and the dopamine agonist utilized (pramipexole) or between EDS and the levodopa equivalent dose. The Cochrane database collects somnolence as a side-effect in placebo-controlled trials of the antiparkinsonian effect of various dopamine agonists, where the somnolence occurred in 21% of patients in the pramipexole group compared with 10% in the placebo group. Other dopaminergic agonists also showed the SED as a side-effect: somnolence occurred in 13% of patients in the pergolide group compared with 4% in the placebo group and in 28% patients in the ropinirole group compared with 6% in the placebo group [26]. However, there are studies that question this symptom, not finding this association of sleepiness with specific drug class [4, 10, 11]. Razmy et al. showed that the mean ESS score did not differ as a function of treatment group, like mean MSLT [11]. The contradiction of these claims justifies the multifactorial nature of EDS. Although an association between the severity of the parkinsonian motor symptoms and pathological somnolence has not been demonstrated, the patients in advanced stages of disease (in clinical status where there is greater structural impairment of the mechanisms responsible for controlling the sleep-wake cycle, involving dopaminergic, noradrenergic, cholinergic, serotonergic, histaminergic, and hypocretinergic neurons) used higher doses and associations of antiparkinsonian medications and they are subject to more adverse effects. Thus, the attempt to define the impact of antiparkinsonian medication isolated on EDS becomes a task more abstract than real.

Moreover, the perception of the nap may be altered in these patients. In an interesting study, Razmy et al. observed patients using high doses of dopaminergic medications, that theoretically had a higher risk for the development of EDS, did not report accurately the presence or absence of daytime sleepiness [11]. The anosognosia of daytime nap is common in patients with PD and in those with disorders of EDS, and it seems to be more severe in patients with PD [27]. These data suggest a more careful evaluation of the ESS scores in this subgroup of patients.

We also observed a considerable amount of patients who met criteria for depression where more than half of the patients have major depressive symptoms. The results are well above the data presented by Brazilian researchers, as Silberman et al., who found a prevalence of depression of 39.1% [28], but within the wide variations in prevalence when using self-assessment questionnaires (27.3 to 76%) [29]. Furthermore, there was a negative correlation between the intensity of depressive symptoms and the presence of excessive sleepiness, where patients with more depressive symptoms were less sleepy. PD patients who may have depressive symptoms that differ from the general population showed significantly less anhedonia but more concentration problems than depressed control subjects [30]. Thus, as the perception of sleep disorders, including excessive daytime sleepiness, may be impaired in depressed PD patients, we should be more careful in evaluating sleep disorders in clinical situations involving high levels of depression. The use of scales for the identification and ranking of depressive symptoms seems to be very valuable in the clinical evaluation of the presence of sleep-related symptoms.

Some limitations were observed in the study. The first is the small number of patients interviewed, which can lead to a restricted analysis of variables related to sleep. The second is the predominance of patients with mild to moderate motor symptoms, with small numbers of patients most severely affected, which may not be representative of the whole. Third, by socioeconomic conditions, objective measures to quantify sleep parameters via polysomnographic have not been carried out. It has been admitted that nocturnal sleep deprivation may contribute to EDS and sleep disorders such as sleep fragmentation, periodic limb movements during sleep and obstructive sleep apnea may contribute to daytime sleepiness in PD. And finally, a specific scale for assessing insomnia was not use. This could be an aid in determining whether the inverse association between excessive daytime sleepiness and depressive symptoms is attributed to greater impact of insomnia in depression or whether there is more misperception of sleep disturbance in more depressed patients. Further studies, using objective measurements of EDS such MSLT, may shed light into this issue.

## 5. Conclusions

Despite these limitations, we conclude that the excessive daytime sleepiness is a common symptom in patients with PD, being more closely associated with the amount of ingested levodopa, and that the presence of depressive symptoms may have a significant impact on the results of self-assessment scales. Therefore, we recommend the assessment of mood changes and the more careful analysis of the results obtained in this specific population of patients with Parkinson's disease.

## Figures and Tables

**Table 1:** Descriptive statistics (*n* = 42).

Measure	Mean ± SD
Age (years)	61.2 ± 11.3
Gender (masculine/feminine)	25/17*
Duration of disease (years)	4.96 ± 3.3
Hoehn and Yahr modified stage	2.1 ± 0.98
Mini-Mental State Examination	26.7 ± 2.6
Epworth sleepiness scale	7.5 ± 4.7
Beck depression inventory	18.7 ± 10.7
Levodopa dosage (mg)	393.45 ± 261.4
Levodopa dosage equivalents (mg)	441.3 ± 272.5

*Total of patients.

**Table 2:** Comparison of variables scores according to excessive daytime sleepiness.

	Epworth < 10 (*n* = 30)	Epworth ≥ 10 (*n* = 12)	*P* (value)
	Mean ± SD	Mean ± SD	
Age (years)	61.5 ± 11.8	60.5 ± 10.4	0.327
Gender (male/female)	11/19*	9/3*	0.594
Duration of disease (years)	5.05 ± 3.1	4.75 ± 3.9	0.157
Hoehn and Yahr modified version	2.1 ± 1.0	2.1 ± 1.0	0.428
Mini-Mental State Examination	26.6 ± 2.8	27.1 ± 2.2	0.089
Beck depression inventory	20.6 ± 10.9	13.8 ± 9.1	0.056
Levodopa dosage (mg)	366.7 ± 228.0	460.4 ± 332.25	0.038
Levodopa dosage equivalents (mg)	410.8 ± 235.1	517.6 ± 348.2	0.46

*Total of patients.

## References

[1] Iranzo A (2006). Parkinson’s disease and sleepiness. *Sleep Medicine Clinics*.

[2] Tandberg E, Larsen JP, Karlsen K (1998). A community-based study of sleep disorders in patients with Parkinson’s disease. *Movement Disorders*.

[3] Gjerstad MD, Aarsland D, Larsen JP (2002). Development of daytime somnolence over time in Parkinson’s disease. *Neurology*.

[4] Hobson DE, Lang AE, Wayne Martin WR, Razmy A, Rivest J, Fleming J (2002). Excessive daytime sleepiness and sudden-onset sleep in Parkinson disease: a survey by the Canadian Movement Disorders Group. *Journal of the American Medical Association*.

[5] Arnulf I, Konofal E, Merino-Andreu M (2002). Parkinson’s disease and sleepiness: an integral part of PD. *Neurology*.

[6] Brodsky MA, Godbold J, Roth T, Olanow CW (2003). Sleepiness in Parkinson’s disease: a controlled study. *Movement Disorders*.

[7] Braga-Neto P, Pereira Da Silva-Júnior F, Sueli Monte F, de Bruin PFC, de Bruin VMS (2004). Snoring and excessive daytime sleepiness in Parkinson’s disease. *Journal of the Neurological Sciences*.

[8] De Cock VC, Vidailhet M, Leu S (2007). Restoration of normal motor control in Parkinson’s disease during REM sleep. *Brain*.

[9] Schlesinger I, Ravin PD (2003). Dopamine agonists induce episodes of irresistible daytime sleepiness. *European Neurology*.

[10] Stevens S, Comella CL, Stepanski EJ (2004). Daytime sleepiness and alertness in patients with Parkinson disease. *Sleep*.

[11] Razmy A, Lang AE, Shapiro CM (2004). Predictors of impaired daytime sleep and wakefulness in patients with Parkinson disease treated with older (Ergot) vs newer (Nonergot) dopamine agonists. *Archives of Neurology*.

[12] Johns MW (1991). A new method for measuring daytime sleepiness: the Epworth sleepiness scale. *Sleep*.

[13] Poryazova R, Benninger D, Waldvogel D, Bassetti CL (2010). Excessive daytime sleepiness in parkinson’s disease: characteristics and determinants. *European Neurology*.

[14] Shpirer I, Miniovitz A, Klein C (2006). Excessive daytime sleepiness in patients with parkinson’s disease: a polysomnography study. *Movement Disorders*.

[15] Bušková J, Klempíř J, Majerová V (2011). Sleep disturbances in untreated Parkinson’s disease. *Journal of Neurology*.

[16] Högl B, Arnulf I, Comella C (2010). Scales to assess sleep impairment in Parkinson’s disease: critique and recommendations. *Movement Disorders*.

[17] Menza M, Dobkin RD, Marin H, Bienfait K (2010). Sleep disturbances in Parkinson’s disease. *Movement Disorders*.

[18] Hobson DE, Lang AE, Wayne Martin WR, Razmy A, Rivest J, Fleming J (2002). Excessive daytime sleepiness and sudden-onset sleep in Parkinson disease: a survey by the Canadian Movement Disorders Group. *Journal of the American Medical Association*.

[19] Kumar S, Bhatia M, Behari M (2003). Excessive daytime sleepiness in Parkinson’s disease as assessed by Epworth Sleepiness Scale (ESS). *Sleep Medicine*.

[20] Ondo WG, Vuong KD, Khan H, Atassi F, Kwak C, Jankovic J (2001). Daytime sleepiness and other sleep disorders in Parkinson’s disease. *Neurology*.

[21] Margis R, Donis K, Schِnwald SV (2008). Psychometric properties of the Parkinson’s Disease Scale—Brazilian version. *Parkinsonism & Related Disorders*.

[22] Santamaria J (2004). How to evaluate excessive daytime sleepiness in Parkinson’s disease. *Neurology*.

[23] Lesser RP, Fahn S, Snider SR (1979). Analysis of the clinical problems in parkinsonism and the complications of long-term levodopa therapy. *Neurology*.

[24] Fabbrini G, Barbanti P, Aurilia C, Pauletti C, Vanacore N, Meco G (2003). Excessive daytime somnolence in Parkinson’s disease. Follow-up after 1 year of treatment. *Neurological Sciences*.

[25] Kaynak D, Kiziltan G, Kaynak H, Benbir G, Uysal O (2005). Sleep and sleepiness in patients with Parkinson’s disease before and after dopaminergic treatment. *European Journal of Neurology*.

[26] Holloway RG, Shoulson I, Fahn S (2004). Pramipexole vs levodopa as initial treatment for Parkinson Disease: a 4-year randomized controlled trial. *Archives of Neurology*.

[27] Merino-Andreu M, Arnulf I, Konofal E, Derenne JP, Agid Y (2003). Unawareness of naps in Parkinson’s disease and in disorders with excessive daytime sleepiness. *Neurology*.

[28] Silberman CD, Laks J, Capitão CF, Rodrigues CS, Moreira I, Engelhardt E (2006). Recognizing depression in patients with Parkinson’s Disease. *Arquives of Neuropsiquiatry*.

[29] Reijnders JSAM, Ehrt U, Weber WEJ, Aarsland D, Leentjens AFG (2007). A systematic review of prevalence studies of depression in Parkinson’s disease. *Movement Disorders*.

[30] Ehrt U, Brønnick K, Leentjens AFG, Larsen JP, Aarsland D (2006). Depressive symptom profile in Parkinson’s disease: a comparison with depression in elderly patients without Parkinson’s disease. *International Journal of Geriatric Psychiatry*.

